# Pectoralis Major Rupture in an Active Female

**DOI:** 10.5435/JAAOSGlobal-D-19-00030

**Published:** 2019-10-16

**Authors:** Mark R. Stringer, Allen N. Cockfield, Thomas R. Sharpe

**Affiliations:** From the Department of Orthopaedic Surgery, Christchurch Hospital, Christchurch, New Zealand.

## Abstract

Pectoralis major rupture was historically a rare occurrence, but the incidence is increasing. Most cases occur from indirect trauma in active men aged 20 to 40 years, especially during bench press. Pectoralis major rupture has never been reported in the literature in a woman in this age group. We report the first case of pectoralis major rupture in a young, active woman who underwent successful surgical fixation.

Pectoralis major rupture was historically a rare occurrence, with 365 reported cases between 1822 and 2010.^1^ However, the incidence is increasing, with 76% of those cases reported since 1990, and several large cohort studies published since that time.^1,2,3,4,5,6,7^ Most cases have occurred in active men in their third to fifth decades, with a small number reported in elderly women.^1,2,3,4,5,6,7^ No case series, cohort study, or meta-analysis has identified any pectorals major ruptures in women under 40 years old. Most cases are caused by indirect trauma, mostly during weight training, specifically during bench press.^1,2,3,4,5,6,7^ Anabolic steroid use was initially not thought to be a major risk factor, but recent studies suggest a high prevalence of steroid use associated with injury.^1,2,4,6,7^

Most patients recognize when pectoralis major rupture occurs, feeling a tearing sensation and often a pop. This is followed by pain, localized swelling, and ecchymosis, with weakness and reduced range of shoulder movement.^8^ Examination findings depend on the location of the tear, but usually include asymmetry of the chest wall and axilla, and weakness of ipsilateral arm adduction and internal rotation.^8^ Assessment with standard radiographs can exclude bony avulsions, fractures, or dislocations.^8^ MRI is the investigation of choice to confirm and evaluate pectoralis major injuries, to characterize the tear location, size, and amount of retraction, and to assist in surgical planning.^2,8^

Nonsurgical management is recommended for some partial tears and proximal tears at the sternoclavicular origin and in elderly or low-demand patients.^2,6,8^ Nonsurgical management includes rest, analgesia, and immobilization in a sling for comfort. Surgical management of any complete tear, including avulsion from the humeral insertion, has better outcomes than nonsurgical management for patient satisfaction, strength, cosmesis, and return to preinjury activity.^3,6,7,8^ Several methods have been described for reattachment of the tendon to the humerus, including transosseous sutures, suture anchors, and cortical buttons.^2,3,4,6,7,8,9^

## Case Report

A 34-year-old right-hand–dominant woman presented to the Christchurch Hospital acute orthopaedic outpatient clinic 1 day after an injury to her right upper arm while playing rugby league. She was preparing to tackle an opponent, when her opponent's head struck her right anterior axilla and upper arm, causing immediate pain in that region. She also developed paresthesia affecting the lateral forearm, which spontaneously resolved soon after. She tried to continue playing, but her right arm was painful, and she was unable to tackle or pass normally, so she voluntarily left the field of play. The next morning she noted ecchymosis over her anteromedial right arm, associated with ongoing pain, so presented for further assessment.

She had previously sustained a right shoulder injury in Switzerland when she fell off a mountain bike approximately 5 years earlier and a traumatic anteroinferior shoulder dislocation when she fell playing rugby 6 months later. She underwent arthroscopic Bankart repair through anterior, lateral, and posterior arthroscopy portals. She reported a full recovery, with no pain or functional limitation before this injury. She takes no regular medications and denied steroid use. She works in a sedentary job. She has two children aged 3 years and 18 months. She smokes casually up to three cigarettes a day. She does not drink alcohol or use recreational drugs. She had recently started cross fit exercise classes but had not performed any chest press exercise.

**Figure 1:**
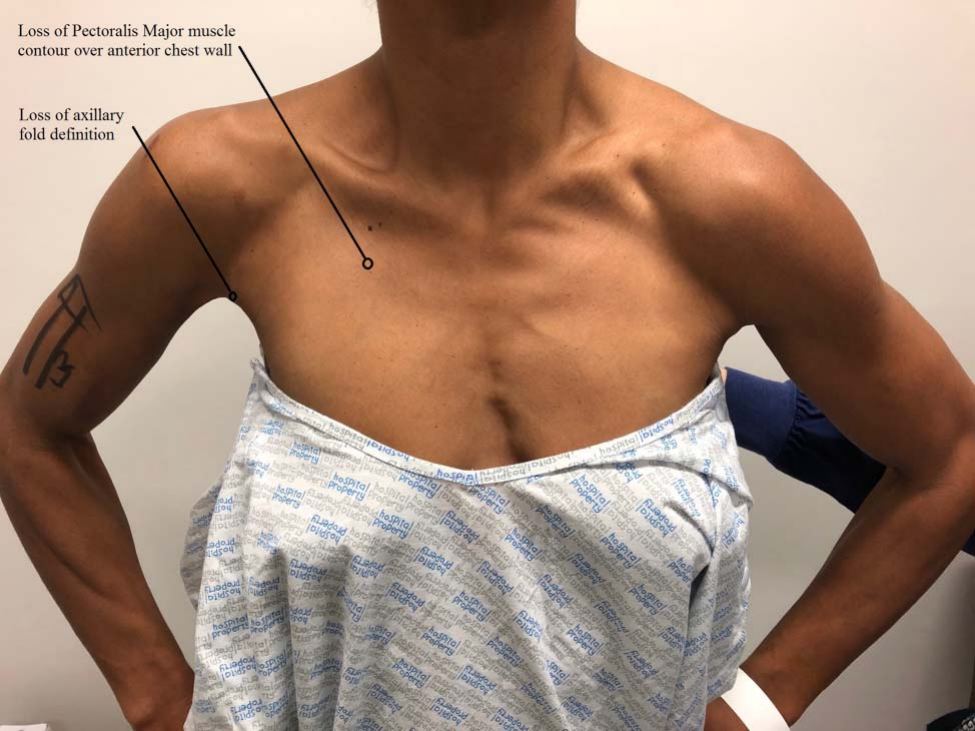
Clinical picture showing loss of muscle contour of the right anterior chest wall and anterior axillary fold.

**Figure 2:**
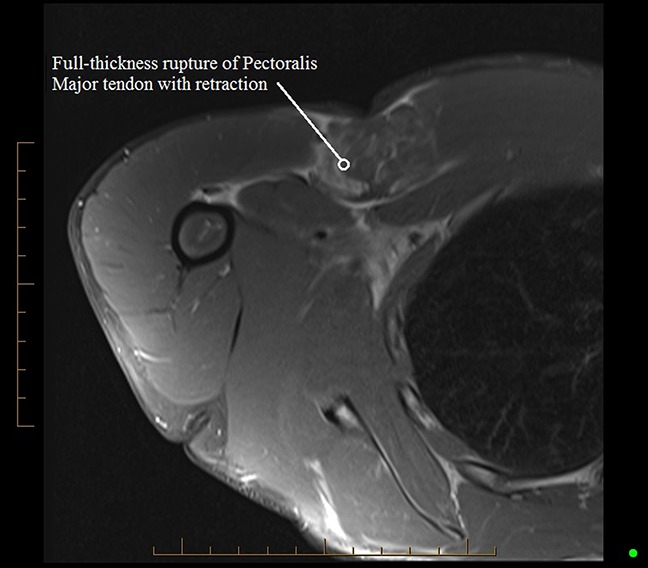
MRI proton density fat-suppressed axial scan right shoulder showing rupture of the pectoralis major tendon at the humeral insertion with 40-mm retraction.

On examination, there was ecchymosis of the anteromedial right arm. There was loss of pectoralis major muscle contour over the right anterior chest wall, with loss of anterior axillary fold definition (Figure 1). There was tenderness over the site of the humeral insertion of the pectoralis major tendon. Shoulder adduction against resistance was painful. There was no associated rotator cuff muscle weakness, and neurovascular examination of the right upper limb was normal. MRI confirmed rupture of the humeral insertion of the pectoralis major tendon with 40-mm retraction (Figure 2).

The patient was offered surgery to reattach the humeral insertion of the pectoralis major tendon, which she accepted. Under general anesthetic in a beach-chair position after intravenous antibiotic prophylaxis, an axillary fold incision was made, and the pectoralis major tendon was identified. Complete avulsion of the humeral insertion was confirmed. The lateral bicipital groove was prepared with a burr, and the tendon reattached with triple whipstitching with size 1 fiber tape and three unicortical 4 mm × 12 mm endobuttons through 4.5-mm drill holes.

**Figure 3:**
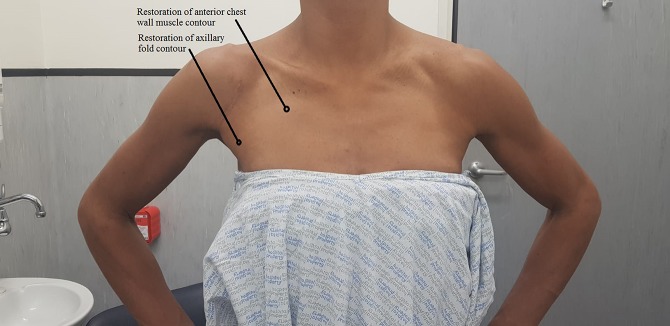
Clinical picture showing restoration of muscle contour of the right anterior chest wall and anterior axillary fold.

**Figure 4:**
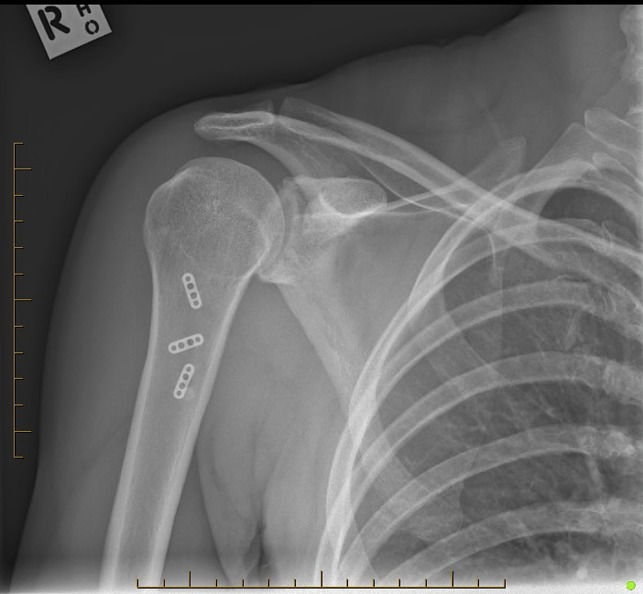
Postoperative AP radiograph.

**Figure 5:**
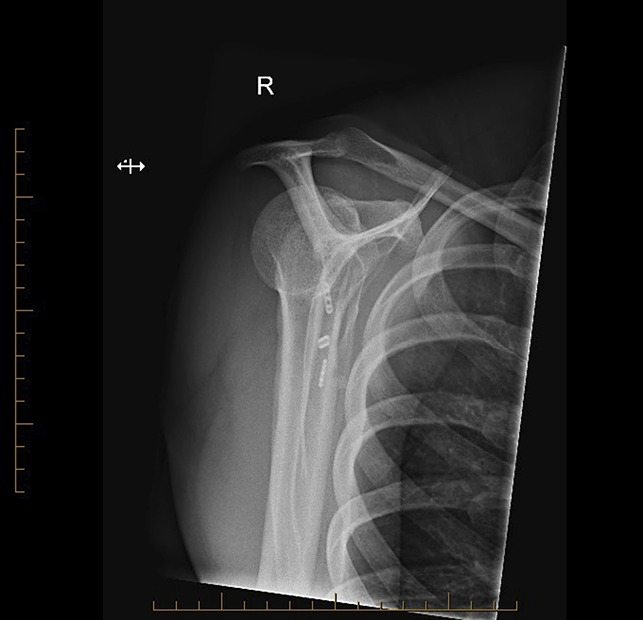
Postoperative lateral radiograph.

Postoperatively, she was treated in a polysling for 6 weeks, with passive mobilization permitted to neutral external rotation and no limitation in forward flexion. At 6-week follow-up, the wound was well healed, with restoration of the normal axillary fold contour (Figure 3). Postoperative AP and lateral radiographs (Figures 4 and 5) were satisfactory, and the patient was cleared to start full active mobilization with the physical therapist. At 3-month follow-up, she had regained full range of active shoulder motion and was cleared to return to weightlifting under the supervision of a physical therapist.

The patient gave full consent to their case being submitted for publication.

## Discussion

Pectoralis major rupture is an uncommon injury, occurring mainly in active men aged 20 to 40 years. There have been no reports of rupture in a woman in this age group. It has rarely been reported in elderly women and has only been reported once in the literature in a middle-aged woman.^1,2,3,4,5,6,7,8,9^ A systematic review by ElMaraghy and Devereaux^1^ identified only 11 cases of pectoralis major rupture in women, and all were elderly aged 73 to 97 years. A case report of a 49-year-old woman, who sustained a complete rupture of the pectoralis major tendon at the humeral insertion while performing a bench press, is notable for her history of bilateral subpectoral breast augmentation 11 years prior and a revision for exchange of implants 5 years before injury. She underwent surgery with transosseous repair. She was able to resume all her functional activities after 6 months.^9^

After their systematic review of all pectoralis major muscle tears between 1822 and 2010, ElMaraghy and Devereaux^1^ proposed a comprehensive classification system based on timing, location, and extent of the tear. The distinction between acute and chronic tears was 6 weeks, with chronic tears often requiring greater exposure and dissection due to adhesions and muscle retraction.^1^ Most tears were found to be at the tendon insertion, followed by the musculotendinous junction.^1,3^ The extent of the tear characterized the thickness and width of the tear.^1^ This classification system can be used to help guide surgical management and allow for meaningful comparison of clinical outcomes.^1^

After this systematic review, a review of the US Military Health System Data Repository identified 291 active-duty military personnel surgically treated for a pectoralis major rupture over a 3-year period from January 2012 to December 2014.^5^ The incidence was 60 per 100,000 person-years. The mean age was 30.5 years, and all patients were men. The majority (64%) were injured while weightlifting. The rupture commonly occurred at the tendinous insertion (39.9%) or myotendinous junction (39.9%). The study did not report any pectoralis major ruptures treated nonoperatively, but the authors believed it would be rare for anyone in this cohort to refuse surgery given the requirement for physical fitness testing, including upper-body strength exercise. Although there were no details on the number and proportion of female active-duty military personnel, this study highlights the absence of any pectoralis major rupture occurring among women, even among a large young, physically active cohort, where a large number of their male counterparts are sustaining this injury.

A prospective randomized study conducted by de Castro Pochini et al^6^ showed superior results from surgical management of pectoralis major ruptures in 60 patients with a mean follow-up of 4 years. All patients were men, with a mean age of 31 years. The bench press exercise was implicated in 48 patients (80%), and 96% were using anabolic steroids. The surgical group had a good or excellent outcome in 88% and a poor outcome in 10%. The nonsurgical group had a good outcome in 27% and a poor outcome in 31%. The notable benefit with surgical management was reinforced in a meta-analysis of 112 cases by Bak et al,^3^ showing the surgical group had good or excellent outcomes in 90% compared with 17% in the nonsurgical group. Furthermore, surgery within 8 weeks had notably better outcomes than delayed surgery, and delayed surgery had better outcomes than nonoperatively treated injuries.^3,4^ Isokinetic evaluation showed a notable 41.7% decrease in strength in the nonsurgical group, compared with a 14.3% decrease in the surgical group.^6^

The rarity of this injury among women is thought to be due to the more elastic nature of their tendons, higher tendon to muscle diameter, and participation in lower energy activities, but there has been no evidence to support this.^4^ The small group of elderly women who sustained this injury was mostly cognitively impaired nursing home residents, fully dependent on care. Pectoralis major rupture was thought to be caused by tearing of stiff, atrophic muscles during transferring, positioning, or dressing.^1,10^ Anabolic steroid use may increase muscle strength disproportionately to the strength of the tendon, insertional site, and musculotendinous junction, resulting in failure under heavy loading.^3^

## Conclusion

Pectoralis major rupture is increasing in incidence and almost exclusively occurs in active men aged 20 to 40 years, usually during bench press. There have been no previous reports of pectoralis major rupture occurring in women in this age group. An MRI scan confirms the diagnosis and assists in surgical planning. Surgical management has superior outcomes to nonsurgical management. This injury can occur in a young active woman and be successfully treated with surgery and supervised rehabilitation.
